# Recombinant Protein-Based Nanoparticles: Elucidating Their Inflammatory Effects In Vivo and Their Potential as a New Therapeutic Format

**DOI:** 10.3390/pharmaceutics12050450

**Published:** 2020-05-13

**Authors:** Laia Gifre-Renom, Estefania Ugarte-Berzal, Erik Martens, Lise Boon, Olivia Cano-Garrido, Esther Martínez-Núñez, Teresa Luque, Ramon Roca-Pinilla, Òscar Conchillo-Solé, Neus Ferrer-Miralles, Antonio Villaverde, Ghislain Opdenakker, Elena Garcia-Fruitós, Anna Arís

**Affiliations:** 1 Department of Ruminant Production, Institut de Recerca i Tecnologia Agroalimentàries (IRTA), 08140 Caldes de Montbui, Spain; laiagifrerenom@gmail.com (L.G.-R.); ramon.roca@irta.cat (R.R.-P.); 2 Laboratory of Immunobiology, Department of Microbiology, Immunology and Transplantation, Rega Institute for Medical Research, KU Leuven, University of Leuven, 3000 Leuven, Belgium; estefania.ugarteberzal@kuleuven.be (E.U.-B.); erik.martens@kuleuven.be (E.M.); lise.boon@kuleuven.be (L.B.); ghislain.opdenakker@kuleuven.be (G.O.); 3 Institut de Biotecnologia i de Biomedicina, Universitat Autònoma de Barcelona, 08193 Cerdanyola del Vallès, Spain; olivia.cano.garrido@gmail.com (O.C.-G.); emartinezn90@gmail.com (E.M.-N.); m3aluque@yahoo.es (T.L.); ocs@bioinf.uab.es (Ò.C.-S.); neus.ferrer@uab.cat (N.F.-M.); antoni.villaverde@uab.cat (A.V.); 4 Networking Research Center on Bioengineering, Biomaterials and Nanomedicine (CIBER-BBN), 08193 Barcelona, Spain; 5 Departament de Genètica i de Microbiologia, Universitat Autònoma de Barcelona, 08193 Cerdanyola del Vallès, Spain

**Keywords:** inclusion body, matrix metalloproteinase-9, *mmp9* knock-out mice, functional nanoparticle, immunogenicity

## Abstract

Bacterial inclusion bodies (IBs) are protein-based nanoparticles of a few hundred nanometers formed during recombinant protein production processes in different bacterial hosts. IBs contain active protein in a mechanically stable nanostructured format that has been broadly characterized, showing promising potential in different fields such as tissue engineering, protein replacement therapies, cancer, and biotechnology. For immunomodulatory purposes, however, the interference of the format immunogenic properties—intrinsic to IBs—with the specific effects of the therapeutic protein is still an uncovered gap. For that, active and inactive forms of the catalytic domain of a matrix metalloproteinase-9 (MMP-9 and mutMMP-9, respectively) have been produced as IBs and compared with the soluble form for dermal inflammatory effects in *mmp9* knock-out mice. After protein injections in air-pouches in the mouse model, MMP-9 IBs induce local neutrophil recruitment and increase pro-inflammatory chemokine levels, lasting for at least two days, whereas the effects triggered by the soluble MMP-9 format fade out after 3 h. Interestingly, the IB intrinsic effects (mutMMP-9 IBs) do not last more than 24 h. Therefore, it may be concluded that IBs could be used for the delivery of therapeutic proteins, such as immunomodulating proteins while preserving their stability in the specific tissue and without triggering important unspecific inflammatory responses due to the protein format.

## 1. Introduction

Recombinant proteins are used for a plethora of therapeutic applications, including cancer therapy, treatment of metabolic disorders, hormone substitution, infectious diseases, thrombolysis, and reproductive disorders, among others [1,2,3,4,5]. During recombinant protein production, bacterial host cells split the overproduced heterologous proteins into soluble and insoluble fractions. The insoluble fraction is also known as inclusion bodies (IBs), which are biophysically described as protein nanomaterials with sizes ranging from 50 to 800 nm, and are easily formed during recombinant protein production [6,7,8]. During many years IBs have been considered a waste product [9]. However, it has been gradually appreciated that IBs may be a promising alternative protein format per se [6,10,11]. Their structure and composition have been extensively characterized evidencing that proteins forming this rough nanomaterial [12,13] are biologically active [6,7,9]. It has also been documented that IBs are mechanically stable nanoparticles with slow protein-release properties, easily produced and isolated through economically affordable processes. It has been reported that they show even higher stability than other protein delivery strategies based on nanoparticles and they also have a better performance in vivo [14]. As a result of IB intrinsic properties, lacking in the soluble and other nanoparticulated formats [14], the applicability of IBs in biotechnology, material sciences, and medical purposes has been explored by different groups [6,10]. The nature of IBs, which combines biological activity and rough surfaces [12,15], allows one to successfully apply these as functional nanoparticles for tissue engineering purposes [16,17]. Another explored application is the injection of targeted-IBs for cancer therapy, proving that these nanoparticles are a stable source of releasing functional proteins [5,18,19]. Besides, it has been shown that IBs can perform an active role as adjuvants for vaccination purposes [20,21], but the real influence of the nanocluster format on inflammatory effects of IBs and their influence on immune-related therapies have never been assessed. Therefore, the exploration of protein-based nanoparticles as a new promising therapeutic format has still some uncovered gaps.

To determine the potential of IBs to deliver an immune-related therapeutic protein in vivo, and to elucidate the possible side-effects of the format on the local inflammatory response, matrix metalloproteinase-9 (MMP-9, also known as gelatinase B) was used as a model protein. MMP-9 is an enzyme that has a relevant role in many biological processes such as wound healing, angiogenesis, reproduction, growth, and tissue development [22] and could be a very relevant drug in the context of several diseases. It is mainly secreted by neutrophils by degranulation and induced in endothelial cells and it is involved in the degradation and remodeling of the extracellular matrix and in chemotaxis [23]. Considering the relevance of MMP-9, we have used a catalytically active fragment of bovine MMP-9 and an inactive mutant of this enzyme produced in *Lactococcus lactis* as IBs [15,24], and we have tested these in a mouse model of skin inflammation. This study demonstrates that the IB format has only a limited inflammatory effect without interference with the specific activity of the model molecule embedded in the protein nanomaterial.

## 2. Materials and Methods

### 2.1. Bacteria Strains and Plasmids

**Figure 1:**
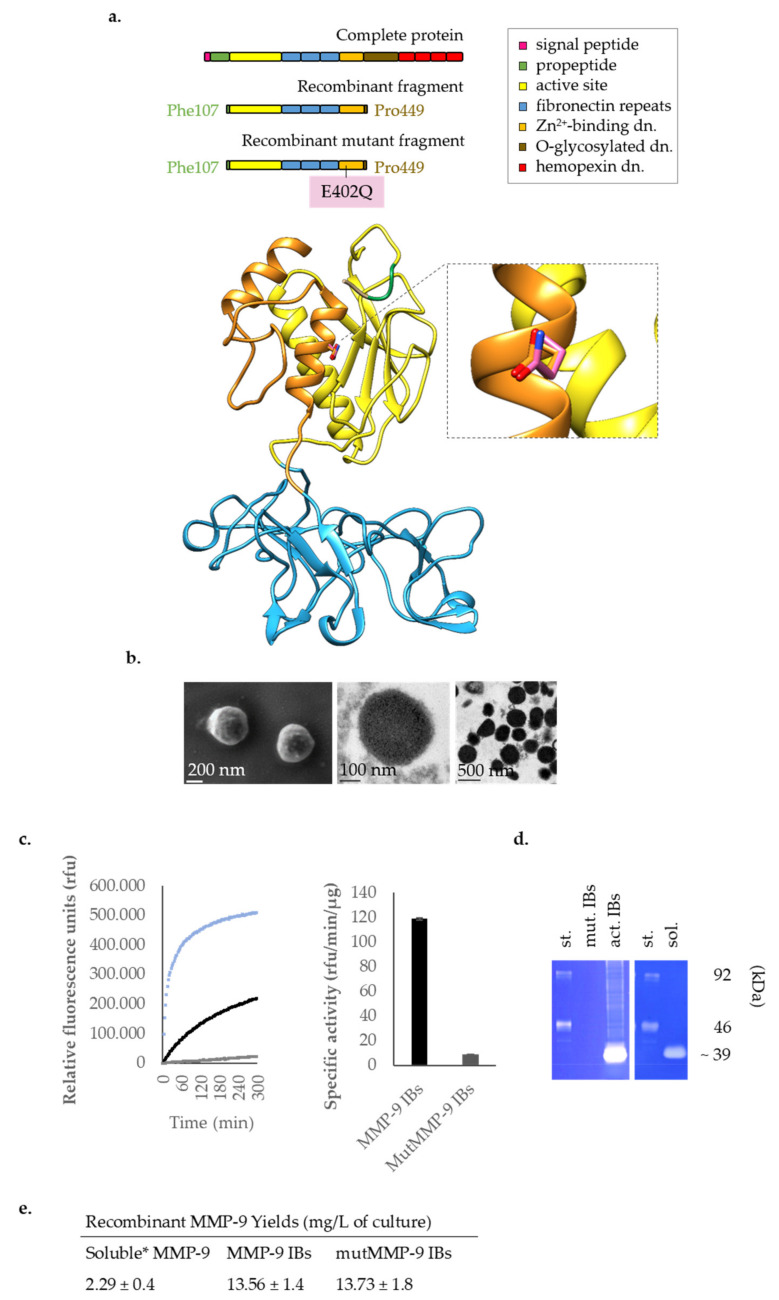
Recombinant MMP-9 and mutMMP-9 structures and functionality. (**a**) Recombinant bovine MMP-9. On top, domain (*dn.*) structure of the complete bovine MMP-9 compared with the recombinantly expressed protein fragment (Phe107–Pro449) and its proteolytic inactive mutant [25]. At the bottom, 3D model for P52176 (Phe107 to Gly444) with the mutated residue E402Q superposed (WT: Glutamic (E) colored in orange, mutant: Glutamine (Q) in purple). Left panel: full view of the generated models. Right panel: detail of the region with the mutated residue that impairs functionality in the mutMMP-9. (**b**) SEM (left) and TEM (middle and right) images of the MMP-9 IBs obtained from *L. lactis*. Recombinant MMP-9 and mutMMP-9 structures and functionality. (**c**) Degradation kinetics for MMP-9 IBs (black), MutMMP-9 IBs (grey), and the soluble MMP-9 (blue) (left) and the corresponding specific activity (SA) for the IBs (right). The soluble MMP-9 SA was 2,282.8 ± 21.9 rfu/min/µg. Error bars indicate standard errors (*n* = 3). (**d**) Zymography analysis showing that MMP-9 degrades gelatin in an electrophoresis gel. *st.* Standard MMP-9 mixture as the marker (see Section 2.8 for details); *mut. IBs.* Mutant MMP-9 IBs; *act. IBs.* Active MMP-9 IBs; *sol.* Soluble MMP-9; *kDa.* kiloDalton. (**e**) Recombinant MMP-9 yields either for soluble and IB forms; * Yield for peak 1 in the chromatography of the solubilized MMP-9; total soluble MMP-9 yield was 4.89 ± 2.8 mg/L of culture.

*Lactococcus lactis* subsp. *cremoris* NZ9000 double mutant Em^R^ ClpP^−^ HtrA^−^ provided by INRA (Jouy-en-Josas, France; patent n. EP1141337B1) was used in this study. A bovine MMP-9 fragment from Phe107 to Pro449 (NCBI, NM_174744.2), which comprises the catalytic, the fibronectin and the zinc-binding domains, was cloned into the plasmid Cm^R^ pNZ8148 (MoBiTech GmbH, Göttingen, Germany) and transformed into competent *L. lactis* ClpP^−^HtrA^−^ as described in Cano-Garrido et al. [15]. A similar MMP-9 cDNA fragment with a single amino acid substitution (E402Q) [25,26], which encodes a proteolytic inactive form of MMP-9 (named mutMMP-9 from now on), was cloned into the pNZ8148 plasmid and transformed into competent *L. lactis* ClpP^−^HtrA^−^. Both genes were C-terminally fused to a His-tag and were codon-optimized for *L. lactis* (Thermo Fisher Scientific, GeneArt GmbH, Regensburg, Germany) [15]. A scheme of the recombinant MMP-9 structure and functionality is provided in Figure 1a.

### 2.2. MMP-9 Modelling

Protein structure homology models for a *Bos taurus* MMP-9 selected region (from F107 to G444) were built with UCSF Modeller 9v13 [27] for wild type (WT) (Uniprot code P52176) and mutant E402Q proteoforms, using the Crystal structure of human MMP-9 (PDB code 1L6J, chain A [28] residues from G105 to G444) as a template. Models were selected according to the best DOPE score [29]. Structure superposition was carried out with ProFit [30], using only the main chain atoms (N,CA,C,O). 

### 2.3. MMP-9 and mutMMP-9 IBs Production in L. lactis

Bacteria were grown without shaking at 30 °C in M17 broth supplemented with glucose (0.5%). Antibiotics were added for plasmid maintenance—chloramphenicol (5 µg/mL) and erythromycin (2.5 µg/mL). Re-inoculated cultures were induced with nisin (12.5 ng/mL) to produce MMP-9 or mutMMP-9 at an OD_600_ of 0.4–0.6, and productions were performed along 3 h. Cultures were recovered and centrifuged at 6,000× *g* for 30 min at 4 °C.

### 2.4. MMP-9 and mutMMP-9 IBs Purification

After recombinant protein expression and recovery of bacteria, each pellet (50 mL) was suspended in sterile PBS (30 mL) and frozen overnight (O/N) at −80 °C. With the following procedure we aimed at the disruption of all bacteria by several freeze-thaw (F/T) cycles and mechanical and chemical procedures. Briefly, thawed bacteria were subjected to 3 rounds of French Press (Thermo FA-078A) at 1,500 psi in the presence of protease inhibitors (Complete EDTA-free, Roche). After a new F/T, samples were incubated for 2 h with lysozyme (0.01 mg/mL) at 37 °C and rotary shaking at 250 rpm. After another F/T cycle, the lysate was incubated for 1 h with Triton X-100 (4 µg/mL) at RT in an orbital rotator shaker. An aliquot of the lysate was plated on agar-M17 broth with glucose (0.5%) and incubated O/N at 30 °C as a first sterility control. Additional F/T cycles were performed until no bacterial colonies were grown on the plates. Next, the lysates were incubated with NP-40 (0.25 µL per mL of sample, 0.25 µL/mL) for 1 h at 4 °C in orbital shaking, followed by 1 h with MgSO_4_ (0.6 µL/mL, stock 1 M) and DNAse I (0.6 µL/mL, stock 1 mg/mL) at 37 °C and 250 rpm. Lysates were centrifuged at 6,000× *g* for 30 min at 4 °C. Each pellet was suspended in lysis buffer (5 mL) with Triton X-100 (0.5%) and F/T. Samples were centrifuged, and under sterile conditions, the supernatant was discarded and pellets were suspended in sterile GIBCO® Dulbecco’s Phosphate-Buffered Saline (DPBS; Gaithersburg, MD, USA) (5 mL). The resulting lysate was aliquoted and centrifuged at 20,000× *g* for 15 min, and the protein pellets were frozen at −80 °C. Some aliquots were diluted in eukaryotic culture media, transferred into the wells of a 96-well plate, and incubated for 48 h at 37 °C and CO_2_ (5%) as a final sterility control. 

### 2.5. Purification of Soluble MMP-9

The soluble MMP-9 fraction and IBs were obtained as described in the work of Gifre-Renom et al. [24]. Briefly, cultures were recovered after 3 h of production and were centrifuged at 6,000× *g* for 15 min at 4 °C. Each pellet from 500 mL cultures was suspended in PBS (30 mL) with protease inhibitors and subjected to 4 rounds of French Press at 1500 psi. Thereafter, lysates were incubated with lysozyme (0.05 mg/mL) for 2 h at 37 °C and 250 rpm orbital shaking. Pellets were obtained by centrifuging the lysates at 15,000× *g* for 45 min at 4 °C and were washed in mQ-H_2_O. After 15 min centrifugation, pellets were weighed and resuspended in solubilization buffer (40 mL/g) containing Tris (40 mM, pH = 8) and *N*-Lauroyl sarcosine (0.2%). The solution was covered with parafilm and incubated on a magnetic stirrer O/N at 4 °C. The obtained solution was centrifuged for 20 min at 20,000× *g* and at 4 °C and the supernatant was collected, filtered, and further purified through Immobilized Metal Affinity Chromatography (IMAC). One mL HiTrap Chelating columns (GE Healthcare, Chicago, IL, USA) were used in an ÄKTA purifier FPLC system (GE Healthcare, Chicago, IL, USA), and both the binding and the elution buffer contained *N*-Lauroyl sarcosine (0.2%). The MMP-9 was eluted by gradually increasing the imidazole concentration until 500 mM, and holding the gradient at each peak to allow peak separation (data not shown). The eluted peaks were dialyzed O/N to PBS at 4 °C with gentle agitation and centrifuged at 15,000× *g* for 15 min at 4 °C to remove possible precipitated protein. The supernatants were filtered under sterile conditions, aliquoted and stored at −80 °C until use.

### 2.6. Protein Determination by Western Blot and Coomassie Blue Staining Analyses

The MMP-9 IBs and mutMMP-9 IBs were quantified by Western blot analysis (ImageJ NIH, version 1.46r, U. S. National Institutes of Health, Bethesda, MD, USA) using a soluble MMP-9 standard. The soluble MMP-9 peaks were quantified by Nanodrop (Thermo Scientific, Waltham, MA, USA) indicating the MMP-9 parameters (MW: 39 kDa and ε: 70,080 M^−1^ cm^−1^; ProtParam-ExPASy, https://web.expasy.org/protparam/). The purity of MMP-9 IBs, mutMMP-9 IBs, and the soluble MMP-9 were analyzed by Coomassie blue staining. 

### 2.7. MMP-9 Activity Determination In Vitro by DQgelatin^TM^ Assay

MMP-9 IBs (10 μg), mutMMP-9 IBs (10 μg), or soluble MMP-9 (10 μg) were plated in a transparent flat-bottom black 96-well plate in triplicate, at a final volume of 150 mL in assay buffer containing CaCl_2_ (5 mM), Tris (50 mM, pH 7.6), NaCl (150 mM), and Tween20 (0.01%). Immediately after adding dye-quenched gelatin (DQgelatin^TM^, Invitrogen, Carlsbad, CA, USA) (0.25 μg per well), the plate was bottom-read every three minutes, O/N, in a fluorescence microplate reader (Victor III multilabel counter, Perkin-Elmer, Waltham, MA, USA) at 495/515 nm (excitation/emission wavelengths). The specific activity of MMP-9 was extracted for each sample from the kinetics data, by obtaining the initial velocity (relative fluorescence units per minute, rfu/min) for each mg of MMP-9 in the wells (rfu/min/μg).

### 2.8. MMP-9 Proteoform Determination in vitro by Gelatin Zymography Analysis

MMP-9 IBs (7 mg), mutMMP-9 IBs (7 mg), and soluble MMP-9 (0.6 ng) were loaded in non-denaturing conditions in SDS-PAGE (10%) gels with gelatin (1%). A standard mix of MMP-9 was loaded as a ladder, containing a multimeric (approximately 250 kDa), a monomeric (92 kDa), and a truncated (46 kDa) form of human MMP-9 [31,32]. After electrophoresis, gels were washed and incubated O/N with developing buffer at 37 °C and were further stained with Coomassie blue in acetic acid (20%), followed by distaining incubations with strong and soft methanol-acetic acid solutions [32].

### 2.9. MMP-9 Injection in Mouse Air-Pouches

A total of sixty C57BL/6 MMP-9 KO mice [33], males and females, were injected intra-dermally on the back with filtered air (3 mL, 0.2 µm filter) on days 0 and 3 to establish the air-pouch compartment, following the standardizations described in the work of Vandooren et al. [34]. Protein samples or DPBS (200 µL) were injected at day 6. Protein injections consisted of MMP-9 (40 µg, soluble or IBs) or mutMMP-9 (40 µg, IBs) in DPBS (200 µL) per injection. The soluble MMP-9 corresponded to the most active fraction (by DQgelatin^TM^ assay) among the different peaks obtained in the chromatography profile (data not shown). At time intervals of 3, 24, and 48 h after injections, mice were euthanized with an intraperitoneal injection of pentobarbital (Dolethal) solution (40 mg/Kg). The exudates in the air-pouches were collected by injecting PBS (2 mL) with heparin (20 U/mL) followed by a gentle massage on the air-pouch and the recovery of all the content. Without taking off the needle, the process was repeated with additional PBS (3 mL) and the total volume was cooled on ice. All experimental procedures were approved by the institutional Ethics Committee (KU Leuven) under license LA1210243 for animal welfare (Project 277/2014, 01-01-2016).

### 2.10. Exudate Cell Count Analysis

The collected exudate volumes were centrifuged at 300× *g* for 5 min in a swing-out cytocentrifuge and supernatants were preserved at −80 °C for cytokine analysis. Cell pellets were washed in ACK buffer (1 mL, GIBCO^®^, Life Technologies) to lyse erythrocytes. After centrifugation, cells were resuspended in PBS and were counted in a Neubauer chamber after trypan blue staining to exclude dead cells.

### 2.11. Flow Cytometry Analysis

Immediately after viable cell counting, we incubated the cells (5 to 10 × 10^5^ cells) in Fc-receptor-blocking antibodies and ZombieAqua-BV510 at RT for 20 min. A master-mix was prepared with the selected antibodies for the characterization of different cell populations: CD11b-APC (monocytes neutrophils, eosinophils, and dendritic cells), CD11c-BV711 (dendritic cells), Ly6C-FITC (monocytes, macrophages and neutrophils) and Ly6G-PE (neutrophils and eosinophils). All the antibodies were purchased from eBioscience (Thermo Fisher Scientific). Cells were incubated with the master-mix (10 µL) for 30–45 min at 4 °C protected from light and were washed twice in PBS. Cells were fixed in formaldehyde (0.37%) and acquired in a fluorescence-activated cell sorting (FACS) Fortessa flow cytometer (BD Biosciences, San Jose, CA, USA). Data were analyzed with the BD FACSDiva™ software v6.1.3.

After compensating the signals for each fluorochrome, live cells excluding doublets were selected. Briefly, cell debris were excluded gating FSC-A^mid-pos^ SSC-A^mid-pos^ quadrant in a dot plot. From this population, singlets were selected drawing a diagonal gate in an FSC-A vs. FSC-H plot excluding outlier events (doublet cells). Afterward, from the last gate obtained, live cells were gated in a new plot selecting the ZombieAqua-BV510-A^neg^ cell population. From these cells, the CD11b-APC-A^pos^ population plotted vs. Ly6C-FITC-A was gated, and this gate was defined as monocytes, macrophages, and neutrophils together (MMN, from now on). From live cell gate, neutrophils were selected gating the Ly6G-PE-A^pos^ CD11b-APC-A^pos^ population, and dendritic cells were selected gating the CD11c-BV711-A^pos^ CD11b-APC-A^neg^ population. Macrophages were selected as the Ly6C-FITC-A^pos^ Ly6G-PE-A^neg^ population. Finally, monocytes were defined as the difference between MMN population and the gates in isolated plots for neutrophils and macrophages. All gates were quantified as a percentage of events from total live cells.

### 2.12. Chemokine ELISA Analysis

The presence of chemokines in the exudate supernatants from the air-pouch assay were analyzed by ELISA (DuoSet ELISA, from R&D Systems, Minneapolis, MN, USA). The chemokines studied correspond grossly to the recruitment of neutrophils (CXCL1/Gro-α/KC and CXCL2/Gro-β/MIP-2α), monocytes and dendritic cells (CCL2/MCP-1), and macrophages (CCL3/MIP-1α). The protocol followed was in accordance with the guidelines provided by the manufacturer, and all washing steps were done twice in PBS containing Tween20 (0.05%). Briefly, flat-bottom 96-well plates were coated with the pertinent capture antibody O/N at 4 °C and washed. Plates were blocked with BSA (0.5%) containing Tween20 (0.05%) for 1 h at RT and washed again. Diluted samples were plated in duplicate and incubated O/N at 4 °C and plates were washed. Plates were incubated for 2 h at RT with the provided biotinylated detection antibodies. After washing, the streptavidin-HRP detection solution was added and incubated for 20 min at 37 °C protected from light. Plates were washed and incubated for 20 min with substrate solution at RT and protected from light, and the reaction was stopped with stop solution provided in the kit. Reads were done in a plate reader at 450 nm and the blank was subtracted from all wells. A sigmoidal standard curve equation and its R^2^ were obtained using the commercial standards provided in each kit.

### 2.13. Statistical Analysis

The animal experiments were performed three times with three mice per condition. Because the experimental variability was limited, data from 3 to 6 animals per condition were pooled. Statistics were carried out with a total of sixty mice. Twelve exudates were obtained after 3 h of protein injection, 24 exudates were collected after 24 h, and 24 exudates after 48 h. Variables were transformed to normalize data when necessary. Data were analyzed using a fixed-effect model (JMP, SAS Institute Inc., Cary, NC, USA). The model included treatment and time and its interaction as main effects, the experiments, and the laboratories where the cytometry data were analyzed as a random effect. Animals were not considered as the random effect as no repeated measures were performed on the same animal. Means and standard deviations represented in graphs correspond to non-transformed data, while *p*-values and asterisks correspond to the Tukey test analyses using transformed data when required. Asterisks depict significant differences to the control group (DPBS). Detailed differences between treatments and time points represented by different letters can be found in the Supplementary Materials (Tables S1–S3).

## 3. Results

Based on structural and functional data, we have produced both active and inactive (or mutant) forms of MMP-9 IBs (MMP-9 IBs [15] and mutMMP-9 IBs, respectively) to evaluate the potential of IBs as a therapeutic and immunomodulatory protein biomaterial. Although both have an almost identical structure [25,26], the substitution of the essential glutamic acid in the Zn^2+^ binding domain for glutamine (E402Q) (Figure 1a) has generated a catalytically inactive MMP-9 variant (mutMMP-9). Soluble MMP-9, MMP-9 IBs, and mutMMP-9 IBs were produced in *Lactococcus lactis* and purified as detailed in Figure S1 [15,24]. The biophysical properties of the IBs format are illustrated in Figure 1b: these consisted of electron-dense nanoparticles that clustered in the host cells. The three MMP-9 recombinant forms (soluble MMP-9, MMP-9 IBs, and mutMMP-9 IBs) were compared for catalysis. As expected, soluble MMP-9 presented higher gelatinolytic activity than MMP-9 in IBs and the mutMMP-9 IBs did not show activity (Figure 1c). These results were corroborated by gelatin zymography analysis (Figure 1d).

**Figure 2:**
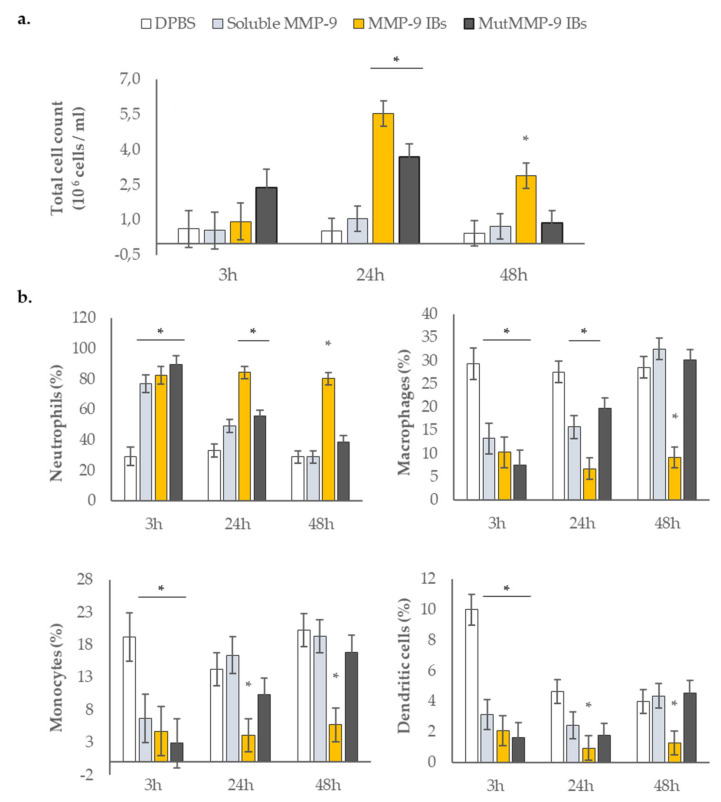
Cellular component analyses in the recovered exudates of the air pouches. (**a**) Total cell counts at 3, 24, and 48 h after injections with the four different treatments. Means and SEM (error bars) from non-transformed data are represented. Asterisks depict significant differences (*p* < 0.005). Detailed differences between treatments and time points represented by different letters can be found in Table S1 in the Supplementary Materials. (**b**) Relative quantification of the different immune cell populations analyzed by flow cytometry. Means and SEM (error bars) from non-transformed data are represented. Asterisks depict significant differences (*p* < 0.0001). Detailed differences between treatments and time points represented by different letters can be found in Table S2 in the Supplementary Materials.

To test the inflammatory capability of the different MMP-9 forms (soluble MMP-9, MMP-9 IBs, and mutMMP-9 IBs), these were injected into air-pouches from *mmp9* knock-out mice, and exudates were recovered at 3, 24, and 48 h to analyze the recruitment of myeloid cells; DPBS was used as the negative control. At 3 h post-injections, no significant differences in total cell counts were observed for any of the MMP-9 treatments (Figure 2a). However, cell recruitment at 24 h was significantly higher in the animals injected with IBs, while soluble MMP-9 treatment did not differ from the control (Figure 2a). Interestingly, after 48 h, the cells recruited in the air-pouches remained high in mice injected with MMP-9 IBs while in mice treated with mutMMP-9 IBs total cell count values returned to basal levels (Figure 2a).

To determine the differences in recruited inflammatory cells, we quantified different cell populations (neutrophils, macrophages, monocytes, and dendritic cells) by flow cytometry (Figure 2b). At 3 h post-injection, all MMP-9-based treatments showed a significant increase in neutrophil percentages compared to DPBS injections (Figure 2b). At longer times (24 and 48 h), only the animals injected with IBs kept neutrophil levels above those injected with DPBS buffer. Both MMP-9 IBs and mutMMP-9 IBs injections increased neutrophil recruitment after 24 h, whereas after 48 h the granulocyte influx was only observed in the MMP-9 IBs treated pouches (Figure 2b). Macrophages, monocytes, and dendritic cells evolved differently from neutrophils, but all three cell populations came with similar profiles (Figure 2b). At 3 h after injections, and for all MMP-9 treatments, a decrease in macrophages, monocytes, and dendritic cells was observed compared to DPBS treatment. No differences were observed for mutMMP-9 IBs treated animals at 24 h and 48 h (Figure 2b). By contrast, exudates from animals treated with active MMP-9 IBs showed a decrease not only in macrophages but also in monocytes and dendritic cells (Figure 2b) after all the time intervals.

**Figure 3:**
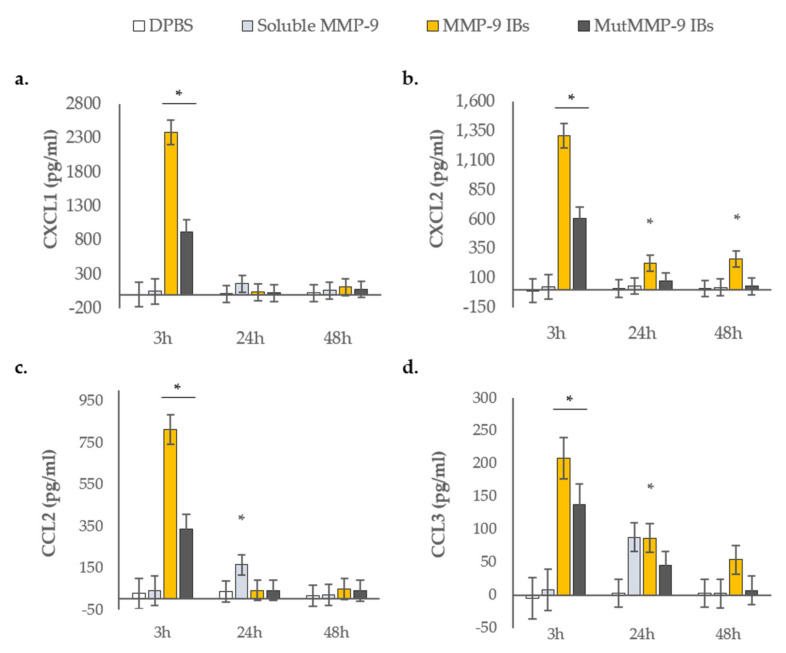
Pro-inflammatory chemokine concentrations in the recovered supernatants of the air pouches. (**a**) CXCL1; (**b**) CXCL2; (**c**) CCL2; (**d**) CCL3. Means and SEM (error bars) from non-transformed data are represented. Asterisks depict significant differences (*p* < 0.0001 in **a**, **b**, and **c**, and *p* < 0.005 in **d**). Detailed differences between treatments and time points represented by different letters can be found in Table S3 in the Supplementary Materials.

To better evaluate whether cell recruitment was a direct effect of the protein biomaterial or an indirect effect through induction of host-derived chemokines for the detected leukocytes, we studied major neutrophil and mononuclear cell chemokines. Supernatants from air-pouch exudates were analyzed to determine the levels of CXCL1 and CXCL2 (chemokines that recruit neutrophils), CCL2 (chemokines for monocytes and dendritic cells) and CCL3 (chemokines for macrophages) (Figure 3). 

At 3 h after injections, both MMP-9 IBs and mutMMP-9 IBs treatments significantly increased CXCL1 levels in comparison with the control and the soluble MMP-9 treated exudates (Figure 3a). In addition, mice treated with MMP-9 IBs showed a 2-fold increase in CXCL1 levels in the air pouch exudates in comparison with those of mice treated with mutMMP-9 IBs (Figure 3a). After 24 and 48 h, no differences were observed for CXCL1 in any of the conditions studied. MMP-9 IBs and the mutMMP-9 IBs had an impact on the levels of CXCL2 at 3 h (Figure 3b), whereas at later time points (24 and 48 h) only the MMP-9 IBs treatment increased CXCL2 levels above the negative control and soluble MMP-9, corroborating the different behavior of MMP-9 IBs compared to mutMMP-9 IBs (Figure 3b). CCL2 profiles showed only increases at 3 h for both MMP-9 IB- and mutMMP-9 IB-based treatments, which decreased after 24 h (Figure 3c). Similarly, CCL3 levels increased in pouches treated with MMP-9 IBs and mutMMP-9 IBs at 3 h in comparison with control and soluble MMP-9 (Figure 3d). After 24 h of injections, only MMP-9 IBs maintained significantly high levels of CCL3 compared to DPBS, while the CCL3 levels after the soluble MMP-9 treatment tended to be greater than those after DPBS injections (Figure 3d). 

## 4. Discussion

Although high purities can be achieved for IBs, meaning that the main content in these particles is the recombinant protein of interest, other impurities such as lipids, RNA and DNA, and other proteins, can be trapped in IBs and are co-purified along with the protein of interest [10]. It has been previously reported that IBs display intrinsic immunostimulant properties that allow these to be used as adjuvants [20]. IBs composed of immunostimulant proteins, such as cytokines, have been reported to provide zebrafish with a greater protective performance in vivo [21]. However, the interference of the intrinsic properties of IBs with the immune performance of IBs has never been elucidated before, and this may be critical when assessing the therapeutic potential for immunostimulant protein-based IBs. For that, in this study, MMP-9 has been chosen as an immunostimulant protein model to address the inflammatory effects in MMP-9-based IBs produced in an LPS-free recombinant system (*L. lactis*). MMP-9 IBs used in this study have been deeply characterized by a physicochemical perspective in a previous study [15], but in vivo behavior in terms of the immune response has not been reported before. Besides chemokines, after tissue damage or a threatening presence, neutrophils, and other leukocytes and endothelial cells secrete MMP-9 as a proenzyme, and this is activated chemically by neutrophil products and proteolytically in the extracellular matrix (ECM) due to the cleavage of the pro-peptide. Then, MMP-9 can degrade ECM components and cell adhesion molecules, increasing the endothelial permeability and facilitating the transmigration of neutrophils and other immune cells to places of tissue damage and promoting an inflammatory response [23]. In this study, we have used a recombinant form of MMP-9 lacking the pro-peptide (Figure 1a) [15], being this immediately able to start the catalysis of ECM components. This MMP-9 form lacks the hemopexin domain that reinforces the binding of tissue inhibitors of metalloproteinases-1 and -3 (TIMP-1, -3) to the enzyme [31,35], making it even more effective in their function in vivo. 

The air-pouch model in mice provides a tool to determine how a foreign substance or formulation thereof influences inflammatory reactions in vivo [34]. Thus, purified IBs (Figure 1b) of MMP-9 and mutMMP-9 (catalytically active and inactive, respectively; Figure 1c,d) were administered into air pouches of a mouse model of skin inflammation [34], and soluble MMP-9 was used as a positive control. By using *mmp9* knock-out mice for this application [33], we aimed to avoid artifacts caused by endogenous mouse MMP-9. At 3 h after the injections into air pouches, all the MMP-9-based treatments (soluble MMP-9, MMP-9 IBs, and mutMMP-9 IBs) showed a significant increase in neutrophil percentages (Figure 2b), in agreement with being the first cell type arriving after a challenge or tissue damage [36]. This indicated a fast effect of MMP-9 in the soluble format, and an inflammatory effect of the IB nanocluster format. With these data, we could not claim that MMP-9 activity in IBs is essential, since no significant differences were observed between MMP-9 IBs and mutMMP-9 IBs (Figure 2b). However, at 24 h, the cellular levels for the pouches treated with the soluble MMP-9 returned to the levels of DPBS treated mice (Figure 2b, excluding macrophages), whereas in mice treated with active MMP-9 IBs the modulation of all the analyzed cell populations remained different. In accordance with a previous article [14], this indicated that the soluble MMP-9 format seems more labile than the MMP-9-based nanoparticles (IBs), for which unspecific effects (carried out by mutMMP-9 IBs) start to disappear yet at 24 h (Figure 2b, except for neutrophils) and are inexistent at 48 h.

Interestingly, there was a strong correlation between early chemokine levels—CXCL1, CXCL2, CCL2, and CCL3—and myeloid cell recruitment (Figure 2 and Figure 3). Firstly, both IBs formats—active and inactive MMP-9 IBs—increased all the analyzed chemokine levels at 3 h while soluble MMP-9 did not (Figure 3). This effect was consistent with the increase in cell recruitment—mainly neutrophils—at 24 h for these treatments (Figure 2b). Secondly, at 24 and 48 h, only in pouches treated with active MMP-9 IBs, an increase in CXCL2 and CCL3 levels was reported (Figure 3b,d), coinciding with the increase in neutrophils at 48 h exclusively for the active MMP-9 IBs treatment (Figure 2b). 

These results are relevant because they elucidated that the recombinant MMP-9 produced in *L. lactis* as IBs [15,24] triggers an inflammatory response due to its degradative capability and not because of the inflammatory response to the nanomaterial (IBs) itself. Thus, while the response triggered by the soluble MMP-9 disappeared after 24 h, the injection of a similar amount of MMP-9 in the IB format extended its effects for more than 48 h, confirming that the IB format provided stability to active MMP-9 [14,37]. Hence, IBs have the potential to be considered in applications such as cancer therapy [5,18,19], vaccination and infectious diseases [4], among others, since the nature of the IB format only transiently stimulates a local and short inflammatory response.

## 5. Conclusions

We report here that the IB format has only a limited inflammatory effect without interference with the specific activity of the model molecule in the protein nanoparticles. In addition, we have shown that the recombinant protein embedded in IBs results in more sustained in vivo effects compared to the soluble (and standard) form. Although more examples will be needed, our work exemplifies the potential of this protein-based nanomaterial as a new and promising delivery tool.

## Supplementary Materials

The following are available online at https://www.mdpi.com/1999-4923/12/5/450/s1, Figure S1: Schematic representation of the protocol used to obtain MMP-9 in *L. lactis* in both soluble and IB formats. Table S1. Detailed output for the statistical analyses in Figure 2a in the main manuscript. Different letters depict differences between different treatments and time points. Table S2. Detailed output for the statistical analyses in Figure 2b in the main manuscript. Different letters depict differences between different treatments and time points. Table S3. Detailed output for the statistical analyses in Figure 3 in the main manuscript. Different letters depict differences between different treatments and time points.

## References

[1] Leader B., Baca Q.J., Golan D.E. (2008). Protein therapeutics: A summary and pharmacological classification. Nat. Rev. Drug Discov..

[2] Sanchez-Garcia L., Martín L., Mangues R., Ferrer-Miralles N., Vázquez E., Villaverde A. (2016). Recombinant pharmaceuticals from microbial cells: A 2015 update. Microb. Cell Fact..

[3] Gifre L., Arís A., Bach À., Garcia-Fruitós E. (2017). Trends in recombinant protein use in animal production. Microb. Cell Fact..

[4] Thwaite R., Berbel C., Aparicio M., Torrealba D., Pesarrodona M., Villaverde A., Borrego J.J., Manchado M., Roher N. (2020). Nanostructured recombinant protein particles raise specific antibodies against the nodavirus NNV coat protein in sole. Fish Shellfish Immunol..

[5] Pesarrodona M., Jauset T., Díaz-Riascos Z.V., Sánchez-Chardi A., Beaulieu M., Seras-Franzoso J., Sánchez-García L., Baltà-Foix R., Mancilla S., Fernández Y. (2019). Targeting Antitumoral Proteins to Breast Cancer by Local Administration of Functional Inclusion Bodies. Adv. Sci..

[6] Rinas U., Garcia-Fruitós E., Corchero J.L., Vázquez E., Seras-Franzoso J., Villaverde A. (2017). Bacterial Inclusion Bodies: Discovering Their Better Half. Trends Biochem. Sci..

[7] Villaverde A., Corchero J.L., Seras-Franzoso J., Garcia-Fruitós E. (2015). Functional protein aggregates: Just the tip of the iceberg. Nanomedicine.

[8] Singhvi P., Saneja A., Srichandan S., Panda A.K. (2020). Bacterial Inclusion Bodies: A Treasure Trove of Bioactive Proteins. Trends Biotechnol..

[9] González-Montalbán N., García-Fruitós E., Villaverde A. (2007). Recombinant protein solubility - does more mean better?. Nat. Biotechnol..

[10] de Marco A., Ferrer-Miralles N., Garcia-Fruitós E., Mitraki A., Peternel S., Rinas U., Trujillo-Roldán M.A., Valdez-Cruz N.A., Vázquez E., Villaverde A. (2019). Bacterial inclusion bodies are industrially exploitable amyloids. FEMS Microbiol. Rev..

[11] Slouka C., Kopp J., Spadiut O., Herwig C. (2019). Perspectives of inclusion bodies for bio-based products: Curse or blessing?. Appl. Microbiol. Biotechnol..

[12] García-Fruitós E., Rodríguez-Carmona E., Díez-Gil C., Ferraz R.M., Vázquez E., Corchero J.L., Cano-Sarabia M., Ratera I., Ventosa N., Veciana J. (2009). Surface cell growth engineering assisted by a novel bacterial nanomaterial. Adv. Mater..

[13] Díez-Gil C., Krabbenborg S., García-Fruitós E., Vazquez E., Rodríguez-Carmona E., Ratera I., Ventosa N., Seras-Franzoso J., Cano-Garrido O., Ferrer-Miralles N. (2010). The nanoscale properties of bacterial inclusion bodies and their effect on mammalian cell proliferation. Biomaterials.

[14] Gifre-Renom L., Seras-Franzoso J., Rafael D., Andrade F., Cano-Garrido O., Martinez-Trucharte F., Ugarte-Berzal E., Martens E., Boon L., Villaverde A. (2020). The biological potential hidden in inclusion bodies. Pharmaceutics.

[15] Cano-Garrido O., Sánchez-Chardi A., Parés S., Giró I., Tatkiewicz W.I., Ferrer-Miralles N., Ratera I., Natalello A., Cubarsi R., Veciana J. (2016). Functional protein-based nanomaterial produced in GRAS microorganism: A new platform for biotechnology. Acta Biomater..

[16] Seras-Franzoso J., Tatkiewicz W.I., Vazquez E., García-Fruitós E., Ratera I., Veciana J., Villaverde A. (2015). Integrating mechanical and biological control of cell proliferation through bioinspired multieffector materials. Nanomedicine.

[17] Seras-Franzoso J., Peebo K., García-Fruitós E., Vázquez E., Rinas U., Villaverde A. (2014). Improving protein delivery of fibroblast growth factor-2 from bacterial inclusion bodies used as cell culture substrates. Acta Biomater..

[18] Unzueta U., Cespedes M.V., Sala R., Alamo P., Sánchez-Chardi A., Pesarrodona M., Sánchez-García L., Cano-Garrido O., Villaverde A., Vázquez E. (2018). Release of targeted protein nanoparticles from functional bacterial amyloids: A death star-like approach. J. Control Release.

[19] Céspedes M.V., Fernández Y., Unzueta U., Mendoza R., Seras-Franzoso J., Sánchez-Chardi A., Álamo P., Toledo-Rubio V., Ferrer-Miralles N., Vázquez E. (2016). Bacterial mimetics of endocrine secretory granules as immobilized in vivo depots for functional protein drugs. Sci. Rep..

[20] Torrealba D., Seras-Franzoso J., Mamat U., Wilke K., Villaverde A., Roher N., Garcia-Fruitós E. (2016). Complex particulate biomaterials as immunostimulant-delivery platforms. PLoS ONE.

[21] Torrealba D., Parra D., Seras-Franzoso J., Vallejos-Vidal E., Yero D., Gibert I., Villaverde A., Garcia-Fruitós E., Roher N. (2016). Nanostructured recombinant cytokines: A highly stable alternative to short-lived prophylactics. Biomaterials.

[22] Vandooren J., Van Den Steen P.E., Opdenakker G. (2013). Biochemistry and molecular biology of gelatinase B or matrix metalloproteinase-9 (MMP-9): The next decade. Crit. Rev. Biochem. Mol. Biol..

[23] Kolaczkowska E., Chadzinska M., Scislowska-Czarnecka A., Plytycz B., Opdenakker G., Arnold B. (2006). Gelatinase B/matrix metalloproteinase-9 contributes to cellular infiltration in a murine model of zymosan peritonitis. Immunobiology.

[24] Gifre-Renom L., Cano-Garrido O., Fàbregas F., Roca-Pinilla R., Seras-Franzoso J., Ferrer-Miralles N., Villaverde A., Bach A., Devant M., Arís A. (2018). A new approach to obtain pure and active proteins from Lactococcus lactis protein aggregates. Sci. Rep..

[25] Rowsell S., Hawtin P., Minshull C.A., Jepson H., Brockbank S.M.V., Barratt D.G., Slater A.M., McPheat W.L., Waterson D., Henney A.M. (2002). Crystal Structure of Human MMP9 in Complex with a Reverse Hydroxamate Inhibitor. J. Mol. Biol..

[26] Roderfeld M., Weiskirchen R., Wagner S., Berres M.L., Henkel C., Grötzinger J., Gressner A.M., Matern S., Roeb E. (2006). Inhibition of hepatic fibrogenesis by matrix metalloproteinase-9 mutants in mice. FASEB J..

[27] Martí-Renom M.A., Stuart A.C., Fiser A., Sánchez R., Melo F., Šali A. (2000). Comparative Protein Structure Modeling of Genes and Genomes. Annu. Rev. Biophys. Biomol. Struct..

[28] Elkins P.A., Ho Y.S., Smith W.W., Janson C.A., D’Alessio K.J., McQueney M.S., Cummings M.D., Romanic A.M. (2002). IUCr Structure of the C-terminally truncated human ProMMP9, a gelatin-binding matrix metalloproteinase. Acta Crystallogr. Sect. D.

[29] Shen M.Y., Sali A. (2006). Statistical potential for assessment and prediction of protein structures. Protein Sci..

[30] Martin A., Porter C. ProFit. http://www.bioinf.org.uk/software/profit/.

[31] Van Den Steen P.E., Van Aelst I., Hvidberg V., Piccard H., Fiten P., Jacobsen C., Moestrup S.K., Fry S., Royle L., Wormald M.R. (2006). The hemopexin and O-glycosylated domains tune gelatinase B/MMP-9 bioavailability via inhibition and binding to cargo receptors. J. Biol. Chem..

[32] Vandooren J., Geurts N., Martens E., Van Den Steen P.E., Opdenakker G. (2013). Zymography methods for visualizing hydrolytic enzymes. Nat. Methods.

[33] Dubois B., Masure S., Hurtenbach U., Paemen L., Heremans H., Van Den Oord J., Sciot R., Meinhardt T., Hämmerling G., Opdenakker G. (1999). Resistance of young gelatinase B – deficient mice to experimental autoimmune encephalomyelitis and necrotizing tail lesions. J. Clin. Investig..

[34] Vandooren J., Berghmans N., Dillen C., Van Aelst I., Ronsse I., Israel L.L., Rosenberger I., Kreuter J., Lellouche J.-P., Michaeli S. (2013). Intradermal air pouch leukocytosis as an in vivo test for nanoparticles. Int. J. Nanomed..

[35] Piccard H., Van den Steen P.E., Opdenakker G. (2007). Hemopexin domains as multifunctional liganding modules in matrix metalloproteinases and other proteins. J. Leukoc. Biol..

[36] De Oliveira S., Rosowski E.E., Huttenlocher A. (2016). Neutrophil migration in infection and wound repair: Going forward in reverse. Nat. Rev. Immunol..

[37] Unzueta U., Seras-Franzoso J., Céspedes M.V., Saccardo P., Cortés F., Rueda F., Garcia-Fruitós E., Ferrer-Miralles N., Mangues R., Vázquez E. (2017). Engineering tumor cell targeting in nanoscale amyloidal materials. Nanotechnology.

