# Supplementary material for: Recombinant Protein-Based Nanoparticles: Elucidating Their Inflammatory Effects In Vivo and Their Potential as a New Therapeutic Format
Source: Pharmaceutics. 2020 May 13;12(5):450. doi: 10.3390/pharmaceutics12050450 (PMC7284881; doi:10.3390/pharmaceutics12050450)
Supplement: Supplementary file 1 [file pharmaceutics-12-00450-s001.pdf]

pharmaceutics

Supplementary Materials: Recombinant Protein-Based Nanoparticles: Elucidating their Inflammatory Effects in vivo and their Potential as a New Therapeutic Format

Laia Gifre-Renom, Estefania Ugarte-Berzal, Erik Martens, Lise Boon, Olivia Cano-Garrido, Esther Martínez-Núñez, Teresa Luque, Ramon Roca-Pinilla, Òscar Conchillo-Solé, Neus Ferrer-Miralles, Antonio Villaverde, Ghislain Opdenakker, Elena Garcia-Fruitós and Anna Arís

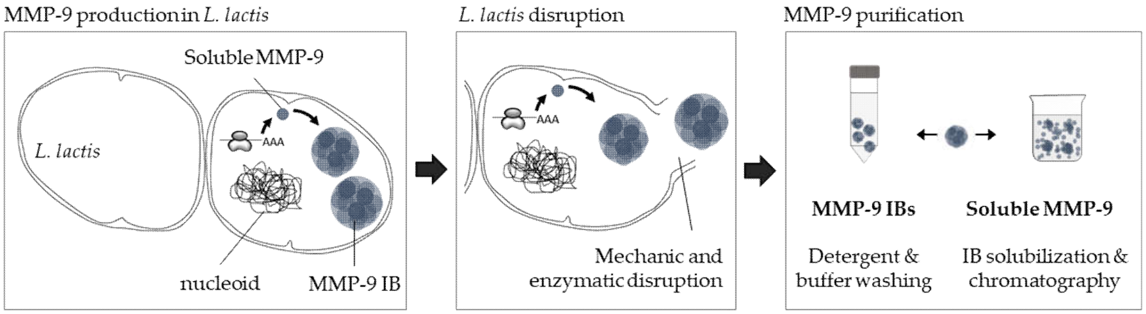

Figure S1. Schematic representation of the protocol used to obtain MMP-9 in *L. lactis* in both soluble and IB format. Recombinant MMP-9 is intracellularly produced under the nisin-controlled gene expression (NICE) system in *L. lactis*. During the overproduction process MMP-9 is mainly accumulated as inclusion bodies (IBs). These protein aggregates are recovered after a mechanic and enzymatic disruption of the bacteria, and IBs are further purified through different detergent and buffer washing steps. The soluble MMP-9 is obtained through the IB solubilization followed by a chromatography purification step, as described in [24].

Table S1. Detailed output for the statistical analyses in Figure 2a in the main manuscript. Different letters depict differences between different treatments and time points.

**Total Cell Count**			
**Heading**	**Hours after Injection**		
	**3**	**24**	**48**
DPBS	b,c,d,e	e	e
Soluble MMP-9	c,d,e	b,c,d,e	e
MMP-9 IBs	b,c,d,e	a	a,b,d
MutMMP-9 IBs	a,b,c,d	a,b	c,e

Table S2. Detailed output for the statistical analyses in Figure 2b in the main manuscript. Different letters depict differences between different treatments and time points.

	**Neutrophils**			**Macrophages**			**Monocytes**			**Dendritic Cells**		
	**Hours after Injection**			**Hours after Injection**			**Hours after Injection**			**Hours after Injection**		
	**3**	**24**	**48**	**3**	**24**	**48**	**3**	**24**	**48**	**3**	**24**	**48**
DPBS	e,f	e,f	f	a,b	a,b	a,b	a,b	a,b,c	a	a	a,b	a,b
Soluble MMP-9	a,b,c	c,d,e	f	c,d	c,d	a	c,d,e,f	a,b,c,d	a,b	b,c	b,c	a,b
MMP-9 IBs	a,b,c	a	a,b	c,d	d	d	d,e,f	e,f	c,d,e	b,c,d	d	c,d
MutMMP-9 IBs	a,b	b,c,d	d,e,f	d	b,c	a,b	f	b,c,d	a,b	c,d	b,c,d	a,b

Table S3. Detailed output for the statistical analyses in Figure 3 in the main manuscript. Different letters depict differences between different treatments and time points.

	**CXCL1**			**CXCL2**			**CCL2**			**CCL3**		
	**Hours after injection**			**Hours after injection**			**Hours after injection**			**Hours after injection**		
	**3**	**24**	**48**	**3**	**24**	**48**	**3**	**24**	**48**	**3**	**24**	**48**
DPBS	c	c	c	d	d	d	c	c	c	d,e	d,e	d,e
Soluble MMP-9	c	c	c	c,d	d	d	c	b,c	c	c,d,e	b,c,d	e
MMP-9 IBs	a	c	c	a	b,c	b,c	a	c	c	a	a,b,c	b,c,d,e
MutMMP-9 IBs	b	c	c	a,b	c,d	d	a,b	c	c	a,b	b,c,d,e	d,e

© 2020 by the authors. Submitted for possible open access publication under the terms and conditions of the Creative Commons Attribution (CC BY) license (http://creativecommons.org/licenses/by/4.0/).
